# FMRP attenuates activity dependent modifications in the mitochondrial proteome

**DOI:** 10.1186/s13041-021-00783-w

**Published:** 2021-04-30

**Authors:** Pernille Bülow, Stephanie A. Zlatic, Peter A. Wenner, Gary J. Bassell, Victor Faundez

**Affiliations:** 1 Department of Cell Biology, Emory University School of Medicine, Atlanta, GA 30322 USA; 2 Department of Physiology, Emory University School of Medicine, Atlanta, GA 30322 USA

**Keywords:** FMRP, Homeostatic plasticity, Mitochondria, Proteomics, Neurodevelopmental disorder, Autism

## Abstract

Homeostatic plasticity is necessary for the construction and maintenance of functional neuronal networks, but principal molecular mechanisms required for or modified by homeostatic plasticity are not well understood. We recently reported that homeostatic plasticity induced by activity deprivation is dysregulated in cortical neurons from Fragile X Mental Retardation protein (FMRP) knockout mice (Bulow et al. in Cell Rep 26: 1378-1388 e1373, 2019). These findings led us to hypothesize that identifying proteins sensitive to activity deprivation and/or FMRP expression could reveal pathways required for or modified by homeostatic plasticity. Here, we report an unbiased quantitative mass spectrometry used to quantify steady-state proteome changes following chronic activity deprivation in wild type and *Fmr1*^*−/y*^ cortical neurons. Proteome hits responsive to both activity deprivation and the *Fmr1*^*−/y*^ genotype were significantly annotated to mitochondria. We found an increased number of mitochondria annotated proteins whose expression was sensitive to activity deprivation in *Fmr1*^*−/y*^ cortical neurons as compared to wild type neurons. These findings support a novel role of FMRP in attenuating mitochondrial proteome modifications induced by activity deprivation.

**Supplementary Information:**

The online version contains supplementary material available at 10.1186/s13041-021-00783-w.

## Introduction

Homeostatic plasticity represents a set of mechanisms that act to maintain physiologically appropriate activity levels within a preset target range or set point. Following chronic activity perturbations, neurons and networks demonstrate the capacity to recover original activity levels by deploying several different adaptive mechanisms [1–6]. While these physiological/functional mechanisms have been extensively studied, far less is known about the underlying molecular cascades that sense, respond, and/or drive these changes. Alterations in cytoplasmic calcium and modifications of protein synthesis are two components of the signaling cascades that underlie these homeostatic mechanisms [4, 7, 8]. Recent evidence has implicated mitochondria as bona fide regulators of neuronal activity. First, by buffering presynaptic calcium levels, mitochondria regulate the neural activity set point for homeostatic plasticity [9]. Secondly, mitochondria provide ATP necessary to support protein synthesis during neuronal maturation and plasticity [10, 11]. Despite these advances, principal mechanisms required for the establishment, modulation and/or maintenance of homeostatic plasticity and the role of mitochondria in this process remain poorly understood.

Several rare human genetic disorders associated with autism and other neurodevelopmental disorders also exhibit impaired homeostatic plasticity [1, 12–16]. This is evident in the case of gene defects in FMR1 and MECP2, causative of Fragile X (FXS) and Rett Syndrome, respectively, whose deficiency alters homeostatic plasticity responses [1, 12, 14, 17, 18]. FMR1 and MECP2 control the expression of a vast number of genes at the translational and transcriptional levels in neurons [19–24]. This suggests that FMR1- or MECP2-dependent gene expression mechanisms likely regulate multiple factors necessary for the establishment, modulation, and maintenance of homeostatic plasticity. 

Here, we sought to identify major pathways or organelles associated with changes in homeostatic plasticity expression. Since we and others reported the necessity of FMRP for the appropriate expression of homeostatic plasticity [1, 12], we hypothesized the existence of common proteins sensitive to both neuronal activity and FMRP expression. To address this hypothesis, we treated wild type and *Fmr1*^*−/y*^ mouse cortical primary neuronal cultures with tetrodotoxin (TTX) plus a NMDA receptor antagonist, and subsequently evaluated their steady state proteomes using unbiased quantitative mass spectrometry. We focused on NMDA blockade as it has been used as a common method of triggering homeostatic plasticity [25, 26]

In contrast with acute proteome labeling strategies performed on wild type neurons, such as BONCAT or SILAC [27–29], we found that following prolonged activity deprivation the mitochondrion, but not the synapse, was the most affected cellular organelle at steady state in both wild type and *Fmr1*^*−/y*^ neurons. While both wild type and *Fmr1*^*−/y*^ neurons displayed changes in the mitoproteome following activity deprivation, these responses were exaggerated in *Fmr1*^−/y^ neurons. Our findings support the idea that mitochondria are modified by activity perturbations, and possibly mediate aspects of homeostatic plasticity expression. We postulate that mitochondria are targets of genes causative of neurodevelopmental disorders and suggest that mitochondrial plasticity defects may contribute to neurodevelopmental disorders and their comorbidities.

## Materials and methods

### Experimental model and subject details

*Mice* *FMR1*^HET^ females (backcrossed on C57BL6 background, B6.129P2-Fmr1tm1Cgr/J Stock No: 003025) were crossed with WT C57BL6 males (Jackson Laboratory) to generate litters of pups with mixed genotypes (*Fmr1*^*−*/y^, Fmr1^HET^ or wild-type (WT)). Thus, for all experiments, *Fmr1*^*−*/y^ male pups were compared to their WT littermate control. We performed PCR to identify genotypes on postnatal day 0–1 (P0-P1) as described previously [30, 31]. The mice were housed in a 12 h light/dark cycle and the animal protocol was approved by the Institutional Animal Care and Use Committees at Emory University.

*Primary cortical neuronal cultures* Cerebral cortices were dissected and cultured from genotyped wild type and *Fmr1*^*−*/y^ pups on P0-P1. The cortices were enzymatically dissociated using trypsin (Thermo Fisher Scientific; 10 min), mechanically dissociated in Minimum Essential Media (MEM; Fisher) supplemented with 10% Fetal Bovine Serum (FBS; Hyclone) and stained to assess viability using Trypan Blue (Sigma). 100,000 neurons were plated on the 15 mm glass coverslip inserts from 35 mm Glass Bottom MatTek petri-dishes (MatTek Corp., Cat no: P35G-1.5-14-C). The glass surface was coated with FBS (Gibco), poly-lysine (Sigma) and laminin (Sigma). The final density in these cultures was 56,000 cell/cm^2^, a density similar to previous studies [29]. The neurons were cultured in standard growth medium (glial conditioned neurobasal (Fisher) supplemented with glutamax (Gibco) and B27 (Invitrogen)), and half of the media was exchanged 2–3 times a week until experimental treatments began. No antibiotics or antimycotics were used. The cultures were maintained in an incubator regulated at 37 °C, 5% CO_2_ and 95% humidified air mix. Cells were cultured as previously described by us [1]. All experiments were performed with days in vitro (DIV) 12 neuronal cultures. This culture and in vitro differentiation parameters were chosen since we previously demonstrated prominent homeostatic plasticity phenotypes under these conditions [1].

### Methods detail

*Proteomics* Cell cultures were placed on ice, washed thrice with PBS and lysed with 8 M Urea (pH 8.5). The urea lysis buffer included an EDTA free HALT protease and phosphatase inhibitor cocktail (Thermo Fisher Scientific, cat no.:78441). The samples were immediately placed on dry ice, and kept at − 80 °C.

Protein Extract Preparation and Digestion. Lysates were quantified by Qubit fluorometry (Life Technologies), 50 μg of each sample was digested overnight with trypsin at room temperature in 12 mM DTT followed by alkylation for 1 h at room temperature in 15 mM iodoacetamide. Trypsin was added to an enzyme: substrate ratio of 1:20. Each sample was acidified in formic acid and subjected to solid phase extraction on an Empore SD C18 plate (3 M catalogue# 6015 SD). Each sample was lyophilized and reconstituted in 140 mM HEPES, pH 8.0, 30% acetonitrile for TMT labeling.

TMT Labeling. 40 μL of acetonitrile was added to each TMT tag tube and mixed aggressively. Tags were incubated at RT for 15 min. 15 μL of label was added to each peptide sample and mixed aggressively. Samples were incubated in an Eppendorf Thermomixer at 300 rpm 25 °C for 1.5 h. Reactions were terminated with the addition of 8 μL of fresh 5% hydroxylamine solution and 15 min incubation at room temperature. Each labeled sample was combined into two experiments, frozen, and lyophilized and subjected to SPE on a High-Density 3 M Empore SDB-XC column (Cat. #4340-HD). The eluent was lyophilized, resuspended and subjected to high pH reverse phase fractionation in a XBridge C18 colum (Waters, part #186003023) on a Agilent 1100 HPLC system equipped with a 150μL sample loop operating at 0.3 mL/min, detector set at 214 nm wavelength.

Mass Spectrometry. Peptides were analyzed by nano LC/MS/MS with a Waters NanoAcquity HPLC system interfaced to a ThermoFisher Fusion Lumos mass spectrometer. Peptides were loaded on a trapping column and eluted over a 75 μm analytical column at 350 nL/min; both columns were packed with Luna C18 resin (Phenomenex). Each high pH RP pool was separated over a 2 h gradient (24 h instrument time total). The mass spectrometer was operated in data-dependent mode, with MS and MS/MS performed in the Orbitrap at 60,000 FWHM resolution and 50,000 FWHM resolution, respectively. A 3 s cycle time was employed for all steps.

Data were processed through the MaxQuant software v1.6.2.3, which served for recalibration of MS data, filtering of database search results at the 1% protein and peptide false discovery rate (FDR), calculation of reporter ion intensities (TMT), isotopic correction of reporter ion intensities (TMT). Data were searched using Andromeda with the following parameters: Enzyme: Trypsin, Database: Swissprot Mouse, Fixed modification: Carbamidomethyl (C), Variable modifications: Oxidation (M), Acetyl (Protein N-term), Fragment Mass Tolerance: 20 ppm. Pertinent MaxQuant settings were: peptide FDR 0.01, Protein FDR 0.01, Min. peptide Length 7, Min. razor + unique peptides 1, Min. unique peptides 0, Second Peptides FALSE, Match Between Runs FALSE. The proteinGroups.txt file was uploaded to Perseus v1.5.5.3 for data processing and analysis. We considered proteins hits as such if the fold of change was above or below (average of all hits in the dataset) ± (2SD) with an alpha < 0.05 as previously described [32, 33].

**Fig. 1:**
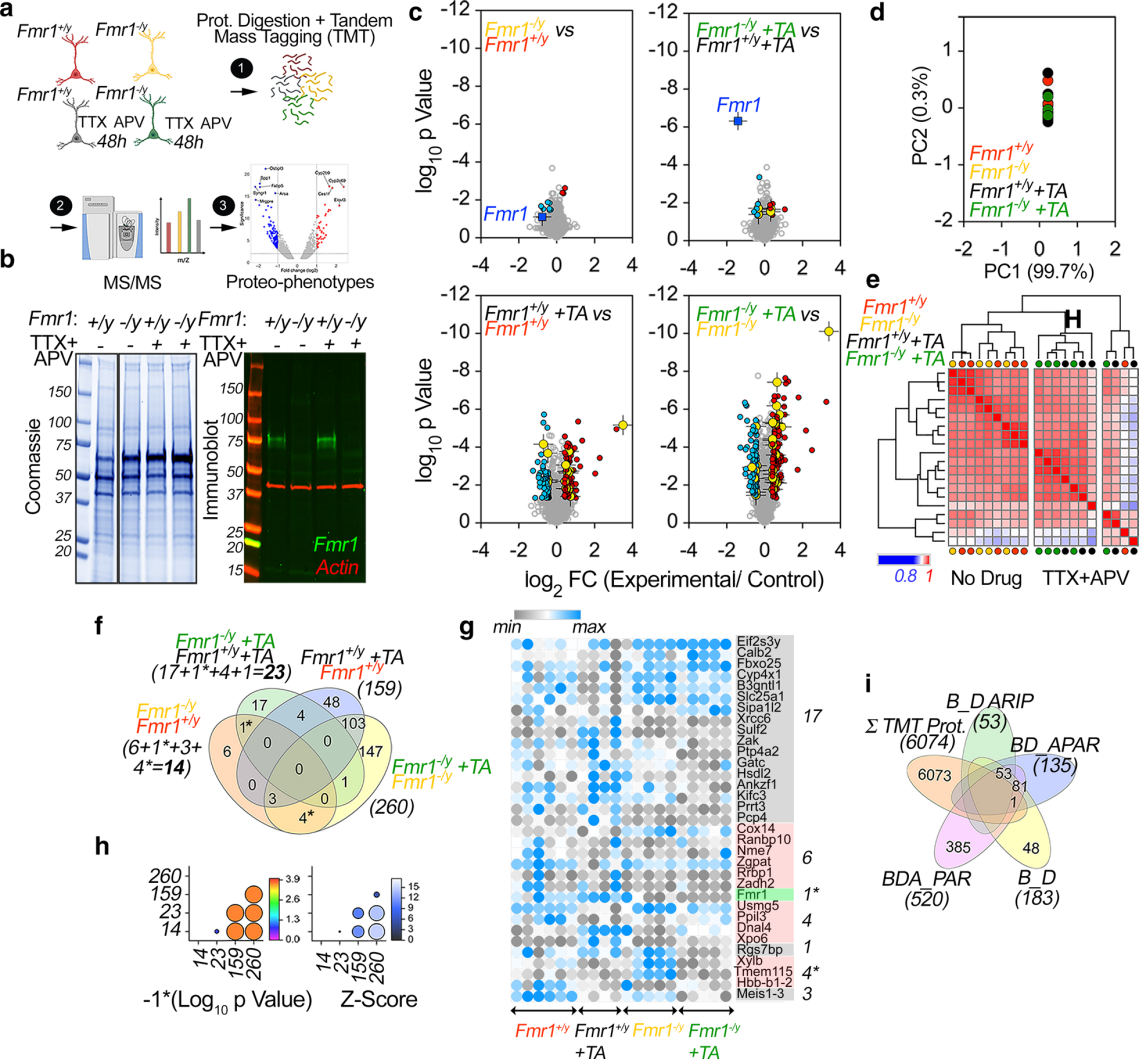
The neuronal proteome sensitive to activity deprivation and *Fmr1*^*−/y*^. **a** Diagram of the experimental design. **b** Representative Coomassie and FMRP-beta actin blots of cultured neuronal extracts. **c** Volcano Plots of TMT mass spectrometry of wild type and *Fmr1*^*−/y*^ neurons (DIV12) incubated in vehicle or TTX-APV for 48 h. N = 6 wild type and n = 4 *Fmr1*^*−/y*^ for vehicle treated cultures, n = 5 cultures for each genotype treated with TTX-APV. The TMT labeling was performed once after all samples were collected. Blue symbols represent down-regulated proteins, red symbols upregulated proteins. FMRP is marked by dark blue and crossed square symbol. Yellow symbols depict mitochondrial proteins significantly changed. **d** Principal component analysis of the 6074 TMT protein quantifications. Note close grouping of all conditions. **e** Similarity matrix of wild type and *Fmr1*^*−/y*^ neurons incubated in the presence of vehicle or TTX-APV for 48 h. Kendall Tau clustering analysis. Note the clustering based on TTX-APV treatment. **f** Venn diagram of common hits in wild type and *Fmr1*^*−/y*^ neurons incubated in vehicle or TTX-APV for 48 h. Parentheses represent total number of proteins significantly modified by treatment and/or genotype. Most protein changes occur after TTX-APV treatment irrespective of genotype. **g** Heat map of the normalized Log2 protein expression levels highlighted in panel (**f**) that compare wild type and *Fmr1*^*−/y*^ cultures (14 hits) and wild type and *Fmr1*^*−/y*^ cultures after TTX-APV treatment (23 hits). **h** *Fmr1*^*−/y*^ neurons incubated with TTX-APV for 48 h significantly increase the number of hits as compared to TTX-APV-treated wild type neurons. P value and Z score analysis of the number of hits in (**f**). X and Y axes numbers represent the number of hits in parentheses in (**f**). Compare wild type TTX-APV (159) to *Fmr1*^*−/y*^ TTX-APV (260), p < 0.0002 and z-score 5. Circle size and color denote p or z-score values. Values calculated with the Vassar’s Difference between Two Independent Proportions tool. **i** Venn diagram all proteins quantified by TMT overlapping with curated the FMRP target mRNAs curated by Suhl et al. 2014. See Additional file 1: Table S1

The mass spectrometry TMT labeling was performed once were each labeling reaction correspond to a biological replicate (see Fig. 1b for details). The mass spectrometry proteomics data have been deposited to the ProteomeXchange Consortium via the PRIDE [34] partner repository with the dataset identifier PXD021473 and 10.6019 /PXD021473.

### Pharmacology

Drugs were used in the following concentrations (in μM): TTX, 1 (Tocris); APV, 100 (Tocris). Drugs were added to fresh standard growth medium and added to the cultures by a complete media change on Day in vitro (DIV) 10 and lasted for 48 h. The treatment drugs were refreshed after 24 h. Control cultures had a simultaneous complete media change but without drugs. Cultures were randomly assigned to each treatment group (control, TTX/APV). All experiments occurred on DIV 12.

### Ontology analyses

We used ENRICHR and ClueGo gene ontology tools to identify annotated cellular compartments as previously described [35, 36]. We used as cutoff for inclusion p values < 0.05. Raw data are presented in Additional file 2: Table S2.

### Statistics

Two tailed statistical tests were performed as indicated in figure legends using http://vassarstats.net/, Kaleida Graph 4.5.2, and Aabel NG2 v5.20. No outlier data curation was applied to statistical analyses.

## Results

### The neuronal proteome is sensitive to activity blockade

We used 12 days in vitro (DIV) primary neuronal cultures from wild type and *Fmr1*^*−/y*^ neurons, and treated them with tetrodotoxin (TTX) and the NMDA receptor antagonist (2*R*)-amino-5-phosphonovaleric acid (APV) for 48 h to induce homeostatic plasticity [1]. To identify proteomic changes affecting whole neurons instead of proteome modifications focalized to the synapse, we focused on steady state changes in the proteome after prolonged changes in neuronal activity. We used Tandem Mass Tagging (TMT) quantitative mass spectrometry to measure proteome modifications [37]. Our steady-state experimental design differs from previously used mass spectrometry approaches where synaptic effects of either activity deprivation or FMRP expression are better revealed with acute proteome labeling [27–29, 38, 39]. In contrast, TMT labeling offers insights into global and long-lasting proteomic changes induced by activity deprivation and FMRP loss. Therefore, the results obtained here to investigate how steady state changes in the *Fmr1*^*−/y*^ proteome might differ from that obtained to measure the dynamics of the newly synthesized proteome and responses to mGluR activation in *Fmr1*^*−/y*^ cells [38].

We quantified 6074 proteins in a 22-plex experimental design to simultaneously compare wild type and *Fmr1*^*−/y*^ neuronal cultures treated with vehicle or TTX-APV (Fig. 1a–c). The proteome discriminated cultures by their FMRP protein expression (Fig. 1c, upper panels blue square symbols and Fig. 1g). Wild type and *Fmr1*^*−/y*^ proteomes were largely similar at baseline except for the expression of a discrete number of non-overlapping proteins (Fig. 1c, upper panels). Principal component analysis (Fig. 1d) and similarity matrix comparisons (Fig. 1e) confirmed the resilience of the steady state proteome to *Fmr1* gene defects. *Fmr1*^*−/y*^ effects on the proteome were discrete; the expression of only 14 proteins was sensitive to genotype, or 0.23% of the quantified proteome (See Fig. 1f and g for list of hits). This result may seem to diverge from the conventional idea that loss of FMRP leads to elevated global protein synthesis, which is often directly measured with acute labeling strategies [38, 40, 41]. However, although a large number of FMRP target mRNAs have increased efficiency of translation, very few of these result in noticeable differences in protein expression at steady state in FMRP deficient neurons [42]. This discrepancy between steady state and acute/dynamic protein levels has been ascribed to increased rates of protein degradation [31] in concurrence with increased protein synthesis. Thus, while steady state levels may appear similar, synthesis and degradation rates of FMRP-targeted mRNAs may differ substantially [42]. The small proteomic difference between genotypes was maintained after TTX/APV treatment: only 23 proteins, or 0.38% of the quantified proteome (See fig. 1f and g for list of hits), differed when comparing activity deprived wild type and *Fmr1*^*−/y*^ neurons (Fig. 1c, f, and h, p = 0.138, z-score 1.48). Notably, the 6,074 quantified proteins poorly overlapped with the FMRP bound mRNAs curated from publicly available independent datasets by Suhl et al. (Fig. 1i) [43]. Only *Fam120a* was shared between our dataset and the 568 gene set curated by Suhl et al. (Fig. 1i). This suggests that the minimal genotype effect in proteome composition may be biased by the low detectability of direct FMRP-binding targets in our dataset.

**Fig. 2:**
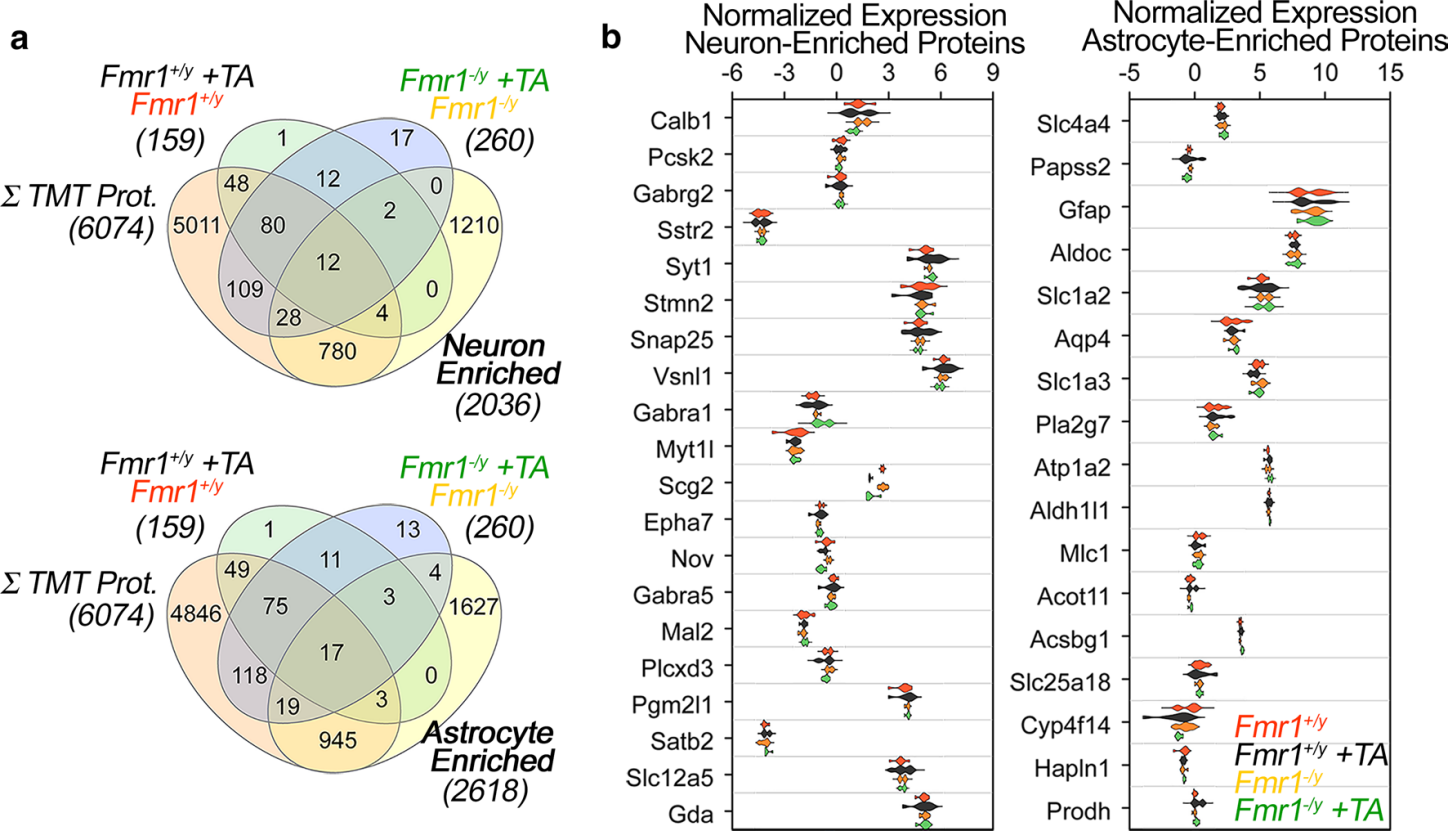
Neuronal and glial markers after activity deprivation and *Fmr1* mutation. **a** Venn Diagram of overlaps between either neuronal or glial markers with all TMT quantified proteins (6074), protein hits sensitive to TTX-APV in wild type cells (159) or *Fmr1*^*−/y*^ cells (260). Neuronal and glia enriched expressed genes were defined according to Cahoy et al. 2008. **b** Violin plots depict TMT normalized expression of most abundant neuronal and astrocyte-enriched proteins in cultures treated with vehicle or TTX-APV both in wild type and *Fmr1*^*−/y*^ genetic backgrounds

In contrast with the genotype effects, the most pronounced proteome modifications happened after TTX-APV treatment. The TTX-APV effect occurred both in wild type and mutant cells (Fig. 1c lower panels, f and g). We identified 159 proteins whose expression changed in wild type cultures after TTX-APV treatment (Fig. 1c lower panels, f and g, and Additional file 1: Table S1). Strikingly, treatment-dependent proteome alterations were significantly more pronounced in *Fmr1*^*−/y*^ cells where we identified a significantly higher number of proteins whose expression was sensitive to TTX-APV treatment: in total 260 proteins were changed in *Fmr1*^*−/y*^ cells after TTX/APV (Fig. 1c lower panels, f, g, and h, p < 0.0002 and z-score of 5.02, and Additional file 1: Table S1). The TTX-APV-induced proteome modifications in wild type and *Fmr1*^*−/y*^ neuronal cultures were mostly overlapping (Fig. 1c upper right panel). In fact, ~ 67% of all proteins sensitive to TTX-APV treatment in wild type cultures were also among the TTX-APV-sensitive proteins in *Fmr1*^*−/y*^ cells (Fig. 1f, 106 protein overlap, and Additional file 1: Table S1). This overlap is 15.5 times higher than expected by chance between these two conditions (p = 6.67e−114, Exact hypergeometric probability). Conversely, ~ 33% of the proteomes sensitive to TTX-APV differed between genotypes. However, gene ontology analysis of this diverging proteome did not identify ontologies unique to TTX-APV treated *Fmr1*^*−/y*^ cells. Unsupervised clustering analysis of the whole proteome confirmed that the main variable segregating proteomes was TTX-APV treatment instead of genotype (Fig. 1e). Genotype and TTX-APV treatment did not change the proportion of neurons and astrocytes in cultured cells as assessed by the expression of neuronal and glial enriched markers (Fig. 2) [44]. These findings demonstrate that TTX-APV treatment preferentially drives proteome modifications in wild type and mutant cultures. However, more proteins were dysregulated following activity deprivation in *Fmr1*^*−/y*^ cells.

### Mitochondrial proteins are enriched in the proteome sensitive to activity blockade in wild type and *Fmr1*^*−/y*^ neurons

**Fig. 3:**
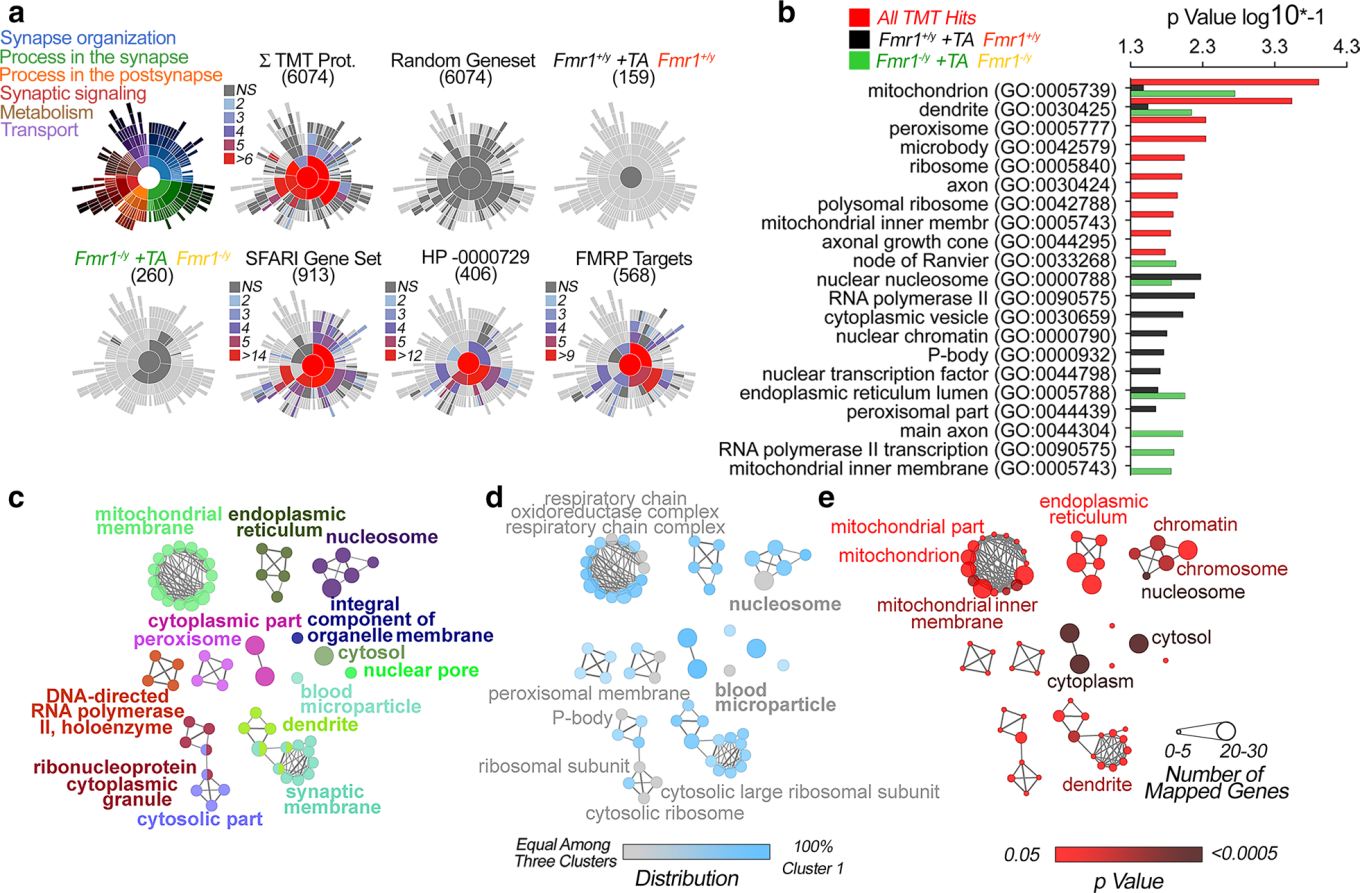
Ontological Analysis of the Proteome Sensitive to Activity Deprivation and *Fmr1*^*−/y*^. **a** Synaptic Ontology Analysis using the SYNGO tool. Sunburst plots represent synaptic annotated GO BP ontologies. Colors represent -log10 FDR corrected p values. Gene sets are defined in Fig. 1. SFARI correspond to the autism spectrum disorder genes curated by https://gene.sfari.org/database/human-gene/. HP-0000729 corresponds to the Human Phenotype Ontology annotated term Autistic behavior HP:0000729 (https://hpo.jax.org/app/browse/term/HP:0000729). **b** GO CC Term ontology analysis performed with the ENRICHR tool. All proteins sensitive to TTX-APV plus those sensitive to the *Fmr1*^*−/y*^ genotype (red bars) were compared to wild type TTX-APV treated hits (black bars) and *Fmr1*^*−/y*^ TTX-APV treated hits (green bars). **c** ClueGO Ontology Analysis of all TMT Hits defined by the red bar in B. Node size represents number of mapped genes. **d** ClueGO Ontology gene percentage contributions among the three gene sets in **b**. **e** ClueGO Ontology Statistics. Non-corrected Two Sided Hypergeometric Test. See Additional file 2: Table S2

We used orthogonal bioinformatic tools to identify pathways and compartments enriched from the hits identified in our proteome datasets. Due to the small size of the genotype-sensitive proteome and the large overlap in TTX-APV sensitive proteome between genotypes, we hypothesized that wild type and *Fmr1*^*−/y*^ neurons could engage similar activity dependent pathways. We first interrogated our datasets for enrichments in proteins annotated to synaptic compartments and processes using the SynGo knowledgebase [45]. The 6,074 proteins quantified in the neuronal cultures significantly enriched pre- and post-synaptic annotated proteins (GO:0,098,793 and GO:0,098,794, p = 5.0E−25 and 1.4E−32, FDR corrected p-value, Fig. 3a). However, the TTX-APV sensitive proteomes did not enrich synaptic annotated genes in either wild type or *Fmr1*^*−/y*^ cells (Fig. 3a). This lack of synaptically annotated genes is comparable to the enrichment obtained with an identically sized randomly generated gene dataset (Fig. 3a). In contrast, the SFARI annotated autism spectrum gene set, the Human Phenotype Ontology autism annotated genes (HP_0000729) [46], as well as the curated list of FMRP-binding mRNAs, all significantly enriched genes annotated to the synapse by the SynGo knowledgebase (Fig. 3a). These findings argue that steady state TTX-APV-dependent changes of the proteome involve compartments other than the pre- and post-synapse.

We used the ENRICHR and ClueGo gene ontology tools to identify annotated cellular compartments within the proteome affected by the TTX-APV treatment in both wild type and *Fmr1*^*−/ y*^ cultures [35, 36]. We predicted that because of the significant overlap between the *Fmr1*^*−/y*^ and wild type TTX-APV proteome data sets (Fig. 1f), they should encompass shared ontologies. We further predicted that pooling together proteins sensitive to TTX-APV in wild type and *Fmr1*^*−/y*^ cultures should additionally enrich relevant cellular compartment ontologies despite increasing dataset size [47]. Conversely, a pooled dataset should decrease the significance of annotated compartments marginally represented in the TTX-APV wild type and TTX-APV *Fmr1*^*−/y*^ datasets [47]. The only annotated terms that satisfied these criteria were the mitochondrion and dendrite (Fig. 3b compare red, black and green bars. GO:0005739, p = 1.22E−04 and GO:0030425, p = 2.88E−04, and Additional file 2: Table S2-1). Mitochondrial and dendrite annotated terms were also identified with the ClueGo algorithm (Fig. 3c–e. GO:0031966, mitochondrial membrane, p = 0.0013. GO:0030425, dendrite, p = 0.0033, and Additional file 2: Table S2-1). Among these compartments, the mitochondrial annotated terms respiratory chain and oxidoreductase complex were similarly represented in each of the TTX-APV-sensitive individual and pooled datasets (Fig. 3d, e, GO:0098803 and GO:1990204, p = 0.048 and 0.046, and Additional file 2: Table S2-1). These results show that mitochondria are robustly identified as compartments affected by TTX-APV in wild type and *Fmr1*^*−/y*^ primary neuronal cultures.

**Fig. 4:**
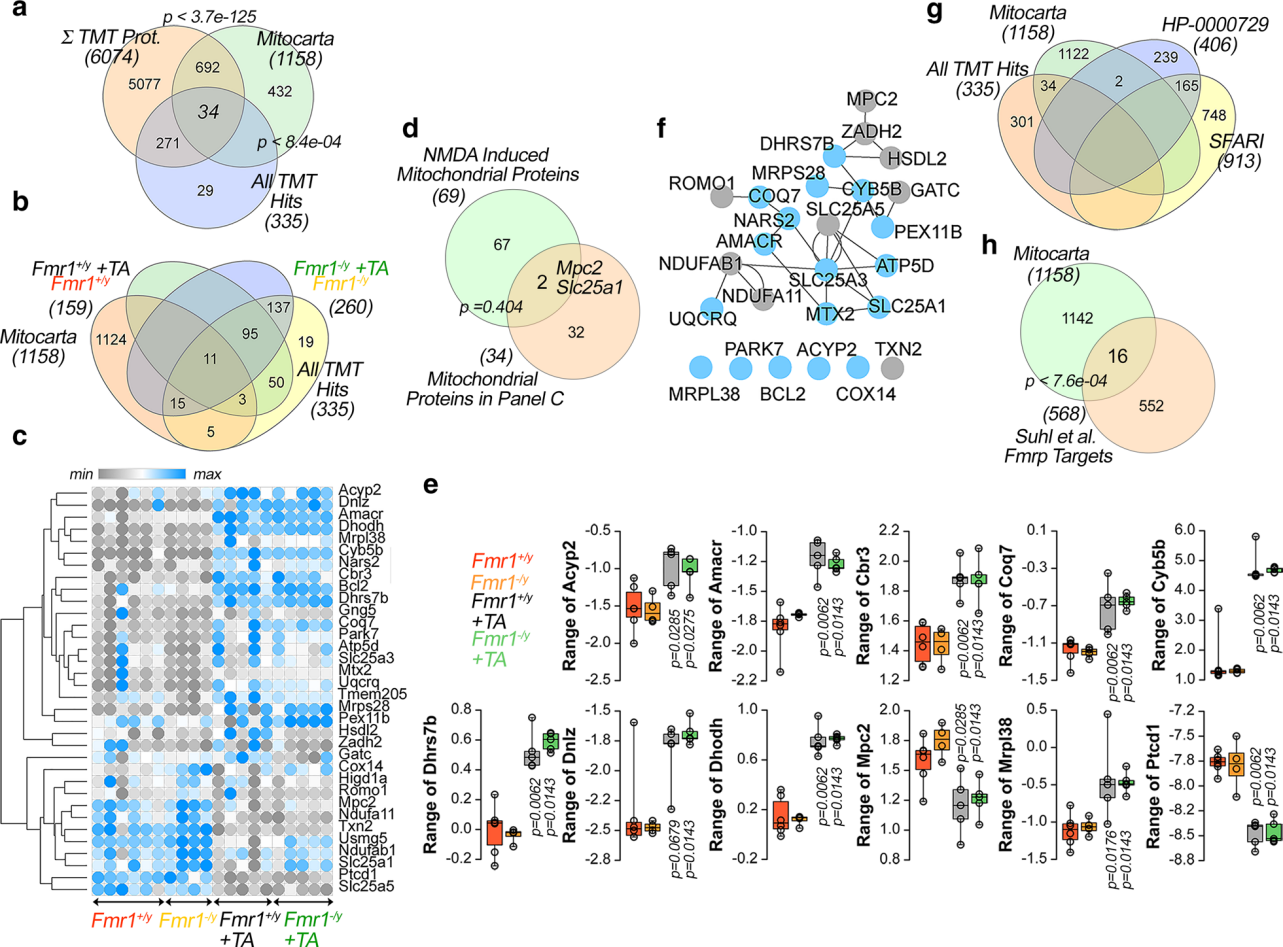
The mitoproteome sensitive to activity deprivation and *Fmr1*^*−/y*^. **a** Venn diagram comparing all Mitocarta 2.0 entries with all TMT quantified proteins (6074), all hits sensitive to TTX-APV plus those sensitive to the *Fmr1*^*−/y*^ genotype (335). **b** Venn diagram comparing all Mitocarta 2.0 mitoproteome with protein hits sensitive to TTX-APV in wild type cells (159) or *Fmr1*^*−/y*^ cells (260) and all TMT proteome hits (335). **c** Hierarchical clustering analysis of the expression of 34 proteins in wild type and *Fmr1*^*−/y*^ cells treated with vehicle or TTX-APV. Kendal Tau clustering analysis of rows. **d** Venn diagram comparing the 34 mitochondrial proteins listed in **c** and those puromycinylated after NMDA treatment of synaptosomes [39]. **e** Normalized TMT Log2 expression of top mitochondrial hits. Kruskal–Wallis Test followed by Mann–Whitney U Test. n = 6 wild type and n = 4 *Fmr1*^*−/y*^ for vehicle treated cultures, n = 5 for each genotype treated with TTX-APV TTX-APV-treated. **f** Interactome of the TTX-APV and *Fmr1*^*−/y*^ sensitive mitoproteomes. Blue nodes represent upregulated proteins. Grey represent downregulated proteins. **g** Venn diagram of all TMT hits, the Mitocarta 2.0 dataset and two autism spectrum disorder curated gene sets. **h** Venn diagram of Mitocarta 2.0 dataset and the Suhl et al. curated Fmrp targets

We next addressed the quality and magnitude of protein changes affecting mitochondria after TTX-APV in wild type and *Fmr1*^*−/y*^ cells. Our proteome covered ~ 63% of all mitochondrial proteins annotated by Mitocarta (Fig. 4a) [48]. The number of mitoproteome hits observed in wild type neurons treated with TTX-APV (11 hits) were increased in the *Fmr1*^*−*/y^ cells (26 hits) (Figs. 1c–h, 3b–e, 4b–e). Among the Mitocarta annotated proteins, 34 were sensitive to treatment with TTX-APV in either wild type or *Fmr1*^*−/y*^ cells (Fig. 1c yellow symbols and Fig. 4a–c). This overlap is 1.8 times higher than expected by chance. The 34 TTX-APV sensitive proteins displayed complex changes in expression: 12 proteins were upregulated after TTX-APV while 22 were downregulated (Fig. 4c, see Additional file 1: Table S1). Of these 34 proteins sensitive to TTX-APV treatment, 11 significantly changed in similar magnitude and direction both in wild type and *Fmr1*^*−/y*^ cells treated with TTX-APV (Fig. 4b, c and e, see Fig. 4c, see Additional file 1: Table S1). Of these 34 mitochondrial proteins, only two hits were shared with a dataset of proteins whose expression is acutely increased after NMDA treatment of synaptosomes, Mpc2 and Slc25a1 (Fig. 4d) [39]. The 34 mitochondrial proteins sensitive to TTX-APV in both genotypes could be assembled into a REACTOME pathway network dominated by the citric acid (TCA) cycle and respiratory electron transport *Homo sapiens* annotated term, which included Ndufa11, Uqcrq, Mpc2, Atp5d, Ndufab1, and Cox14 (Fig. 4c, e, and f. R-HSA-1428517, p = 2.05E−7) [49]. While these proteins are annotated to a citric acid (TCA) cycle ontological term, they did not overlap with proper citric acid (TCA) cycle enzymes identified previously in *Fmr1*^*−/y*^ synaptosomes [50]. The functional pathway network composed by the 34 mitochondrial proteins included proteins up- and down-regulated after TTX-APV treatment (Fig. 4f, teal and gray nodes, respectively) precluding precise metabolic hypothesis formulation from these changes in the mitochondrial proteome.

Since mitochondrial ontologies were revealed by their TTX-APV treatment rather than the *Fmr1* genotype, we further inquired whether mitochondrial genes were annotated either to autism spectrum disorder curated databases or whether mitochondrial nuclear encoded mRNAs were bound by FMRP. Only two Mitocarta genes were present in the HPO annotated autism spectrum disorder term, an overlap 10 times below expected (Fig. 4g, HP_000072) [46]. Moreover, only 16 mRNAs annotated to Mitocarta were targets of FMRP, an overlap twofold lower than expected (Fig. 4h, p < 7.6e−04). These results suggest two different possible models. First, *Fmr1* mutations and other autism spectrum disorder gene defects may indirectly regulate mitochondrial protein composition at steady state. Since FMRP targets are known to be linked to synapse function, and synapses regulate mitochondria function, the dysregulation we observe in mitochondrial proteome in *Fmr1*^*−/y*^ cells, could be downstream of synaptic FMRP targets. Alternatively, *Fmr1* gene defects may directly affect mitochondria, but are below detection and/or exist in a compartment specific manner rather than by globally altering mitochondrial composition in neurons. Compartmentally localized mitochondrial changes could evade detection by mass spectrometry analysis of whole culture lysates.

## Discussion

Here, we sought to identify novel mechanisms required or modified during the induction, establishment and/or maintenance of homeostatic plasticity. To this end, we comprehensively and unbiasedly quantified steady state proteomic modifications in wild type and *Fmr1*^*−*/y^ mouse primary cortical neurons following activity deprivation with TTX-APV (48 h, Fig. 1a). We selected TTX-APV and FMRP expression because we previously showed that this drug treatment induced homeostatic intrinsic plasticity in a functionally altered manner in *Fmr1*^*−/y*^ neurons [1]. We draw two conclusions: first, the mitochondrial proteome is the most affected during chronic activity deprivation in a steady-state analysis. This finding represents a conceptual advance since a link between homeostatic intrinsic plasticity and the mitoproteome has not been previously shown. Second, *Fmr1*^*−/y*^ neurons display exaggerated changes in the mitoproteome during activity deprivation. Thus, FMRP attenuates activity dependent modifications of the mitoproteome.

Our findings converge with those of Licznerski et al. (2020) in the preferential compromise of the mitochondrial proteome in *Fmr1*^*−/y*^ neurons [50]. However, our results capture complementary mitochondrial proteomes as our datasets include different subunits of complex V (Atp5d) and proteins annotated to a REACTOME Krebs cycle ontology [50]. In fact, we found decreased oxygen consumption sensitive to oligomycin, an indication of complex V activity, as well as decreased basal respiration in cultured cortical *Fmr1*^*−/y*^ neurons (unpublished data) paralleling some of the findings by Licznerski et al. in synaptosomes. We suspect that the acute labeling of synaptosome proteomes with puromycin by Licznerski et al. (2020) may explain in part the mitochondrial proteome complementarity between datasets (see below).

How does FMRP deficiency trigger enhanced changes in the mitochondrion proteome during TTX/APV? We speculate that FMRP does so by an indirect mechanism. This assertion is founded on the marked underrepresentation of the mitochondrial proteome among the nuclear encoded mRNAs bound and translationally regulated by FMRP (Fig. 4g). In fact, only 16 of the 1158 Mitocarta annotated mitochondrial proteins are FMRP targets [19–21, 43, 51]. While this is a small number, these 16 proteins could have widespread impacts in the mitochondrial proteome. An interesting candidate for an indirect FMRP-dependent mechanism controlling the mitochondrial proteome is the mitochondrial ribosome [52]. We recently found that downregulation of mitochondrial ribosome subunits phenocopies mutant dFmr1 phenotypes in *Drosophila* synapses [53–55]. We posit that this mechanism is indirect because none of the ~ 80 mitochondrial ribosome mRNAs are among the curated FMRP targets [43]. mRNAs encoding mitochondrial ribosome subunits are increased in FMR1^−/y^ neuronal cells, yet their translational efficiency is decreased [52]. FMRP is best known as a translational suppressor [41], but it also regulates protein conformation/function directly or activates mRNA translation [56, 57]. FMRP can also directly bind to the cytoplasmic ribosome affecting the expression of mitochondrial proteins encoded in the nuclear genome [58]. Any of these mechanisms could indirectly impair the function and/or the composition of the mitochondrial ribosome. A compromised mitochondrial ribosome could account for altered mitochondrial functions observed in *Fmr1*^*−*/y^ cultures [50, 52, 59–65]. Alternatively and/or in addition, we know that loss of FMRP dysregulates synaptic and ion channel protein expression at baseline, and these alterations may lead to altered mitochondrial plasticity during activity deprivation. Since FMRP directly regulates the synaptic proteome via translational control, and directly regulates ion channel function through RNA and protein associations, this opens up numerous possibilities on the underlying mechanism for altered mitochondrial plasticity in FXS.

We focused on the consequences of prolonged activity deprivation on the steady state proteome. Our findings complement proteome analyses studying protein synthesis and half-life in primary cultured neurons using non-equilibrium proteome labeling strategies, such as puromycinylation of the proteome, BONCAT or isotopic labeling of neurons or neuronal fractions either by pulsed methionine, pulsed SILAC, or SILAC pseudoequilibrium labeling [27–29, 38, 39, 50, 66]. Puromycylated, SILAC, and BONCAT acute labeled proteomes are enriched in synaptic proteins, a fact that highlights the responsiveness of the pre- and postsynaptic proteome to rapid changes in neuronal activity. In contrast, our findings indicate that the presynaptic and postsynaptic proteomes at the steady state remain mostly unchanged after prolonged activity deprivation both in wild type and *Fmr1*^*−*/y^ neurons (Fig. 4). Our activity-dependent steady-state proteome is enriched in proteins annotated to mitochondrial ontologies (Fig. 4b–e). The apparent discrepancy between the results obtained with acute labeling of the proteome and our steady state changes cannot be attributed to an underrepresentation of the synaptic proteome in our dataset, as they were significantly enriched (Fig. 4a). Thus, we postulate that the following variables could contribute to the ontological differences between acute and steady-state proteomes after activity deprivation. First, the duration or the pharmacological agents used to achieve activity deprivation could underlie differences. We used 48 h of TTX-APV treatment in contrast with the 24 h of TTX treatment used for acute proteome labeling [27, 29]. This time difference may play an important role as the half-life of synapse annotated proteins is ~ 5 days, while proteins annotated to the mitochondria possess a half-life between ~ 9 and 15 days [28]. Thus, acute labeling strategies are more likely to identify changes in the synaptic proteome and other cellular compartments with shorter half-lives. Further, the addition of an NMDA receptor blocker could contribute to differences as this is known to be critical for the induction of homeostatic plasticity [25, 26]. A second variable is the difference in neuron species. While we used postnatal mouse cortical neurons, others have used postnatal hippocampal rat neurons [27–29, 47, 67]. A third elements is the degree of differentiation in vitro. We chose 12 DIV neuronal culture conditions as this experimental design revealed to us homeostatic intrinsic plasticity phenotypes in *Fmr1*^*−*/y^ neurons [1]. However, our conditions differ from other studies where cells were cultured for 18 to 21 DIV. These DIV differences could contribute to the non-overlapping proteome findings [28, 29, 66]. Finally, compartment-specific mitochondrial responses could produce differences. This hypothesis is supported by evidence indicating that changes in mitochondrial protein expression following depolarization can only be detected in synaptic-enriched fractions [39]. We postulate that our activity- and *Fmr1*-dependent steady-state proteome reflects long-lived compartmental changes rather than rapid activity-dependent remodeling of acute proteome labeling. Further, we hypothesize that FMRP plays an important role in attenuating the activity deprivation induced changes in the mitochondrial proteome.

## Supplementary Information

**Additional file 1: Table S1.** Proteome hits sensitive to activity deprivation and *Fmr1*^*−/y*^ quantified by TMT mass spectrometry.

**Additional file 2: Table S2.** Ontological analyses of the proteome sensitive to activity deprivation and *Fmr1*^*−/y*^.

## Data Availability

The mass spectrometry proteomics data have been deposited to the ProteomeXchange Consortium via the PRIDE with the dataset identifier PXD021473 and 10.6019 / PXD021473.
